# Magnitude of Thyroid Disorders in Patients with Abnormal Uterine Bleeding

**DOI:** 10.7759/cureus.118064

**Published:** 2026-10-17

**Authors:** Sankalpa Addamane Jayachandra, Jayanthy T

**Affiliations:** 1 Obstetrics and Gynaecology, University Hospitals of Leicester NHS Trust, Leicester, GBR; 2 Obstetrics and Gynaecology, East Point College of Medical Sciences &amp; Research Centre, Bangalore, IND

**Keywords:** abnormal uterine bleeding, abnormal uterine bleeding (aub), aub (e/o), hyperthyroidism, hypothyroidism, menorrhagia, polymenorrhea

## Abstract

Background: Abnormal uterine bleeding (AUB) is a common gynaecological complaint, and thyroid dysfunction may contribute to menstrual abnormalities. This study evaluated the prevalence of thyroid dysfunction among women presenting with AUB and its association with clinical characteristics.

Methods: This observational study was conducted in the Department of Obstetrics and Gynaecology, Kempegowda Institute of Medical Sciences, Bengaluru, India, from January 2020 to June 2021. It included 100 women with AUB. Demographic and clinical data, such as age, parity, body mass index (BMI), type and duration of AUB, thyroid status, family history, and goitre and thyroid-related symptoms, were documented. Thyroid function tests were used to group the participants into the following categories: euthyroid, subclinical, hypothyroid, and hyperthyroid.

Results: The mean age was 34.85 ± 8.29 years, and the mean BMI was 24.74 ± 3.36 kg/m². Thyroid dysfunction was found to be present in 20% of the participants. Heavy menstrual bleeding was the most common AUB pattern (40%), which was not significantly associated with thyroid status (p = 0.398). Significant associations were observed between thyroid status and family history (p = 0.045), goitre (p < 0.001), and thyroid-related symptoms (p < 0.001).

Findings: Thyroid dysfunction was found to be relatively common in women with AUB. Women with AUB may need to be evaluated for thyroid function, especially if they experience symptoms or signs suggestive of thyroid dysfunction.

## Introduction

Abnormal uterine bleeding (AUB) is a common gynaecological problem among women of reproductive age and may result from structural or non-structural causes. The International Federation of Gynecology and Obstetrics (FIGO) classifies AUB using the PALM-COEIN system, which includes ovulatory dysfunction among its non-structural causes. Ovulation disorders may result in irregular or excessive uterine bleeding and may be associated with endocrine abnormalities, including thyroid dysfunction [1].

Thyroid hormones play an important role in female reproductive physiology. Multiple points of interaction exist between the anterior and posterior, pituitary and the hypothalamic-thyroid axis and the hypothalamo-pituitary-ovarian axis, with thyroid dysfunction affecting gonadotropin secretion, ovarian function, sex hormone metabolism, and/or menstrual cyclicity [2,3]. Further, human oocytes, cumulus cells, and granulosa cells express thyroid hormone receptors, suggesting a direct action of thyroid hormones on the ovary [4]. Both hypothyroidism and hyperthyroidism are associated with menstrual disturbances. Heavy or prolonged menstrual bleeding is more commonly reported with hypothyroidism, while oligomenorrhoea, hypomenorrhoea, or amenorrhoea is reported with hyperthyroidism [2,5]. The frequency of thyroid dysfunction among women with AUB varies between populations, although subclinical hypothyroidism and hypothyroidism are commonly reported abnormalities [6-9]. 

Menstrual abnormalities may occasionally precede the development of the overt clinical features of thyroid disease. Therefore, the identification of thyroid dysfunction in women with otherwise unexplained AUB may help to ensure proper management and avoid unnecessary interventions [2,5,6]. The objective of the current study is to evaluate the extent and type of thyroid dysfunction in women of reproductive age (15-45 years) with AUB related to ovulatory dysfunction (Endometrial/Ovulatory (E/O)). This study analysed the distribution of menstrual abnormalities based on thyroid status and the association of thyroid dysfunction with selected clinical abnormalities, such as body mass index (BMI), family history of thyroid disease, goitre, and thyroid-related symptoms. The research aimed to determine the prevalence of thyroid disorder in women with AUB and assess its association with clinical characteristics.

## Materials and methods

This was a hospital-based observational cross-sectional study conducted in the Department of Obstetrics and Gynaecology, Kempegowda Institute of Medical Sciences and Research Centre, Bengaluru, Karnataka, India. The study was conducted over a period of 18 months, from January 2020 to June 2021. Women aged 15-45 years presenting to the gynaecology outpatient or inpatient department with AUB (E/O) were considered for participation. Participants were recruited using a purposive sampling technique, and a total of 100 women were included in the study. The study was approved by the Institutional Ethics Committee of Kempegowda Institute of Medical Sciences and Research Centre, Bengaluru, under approval number KIMS/IEC/D079/2019. Informed consent for participation and open-access publication was obtained or waived from the participants, as applicable.

The study protocol included a thorough history taking regarding age, bleeding pattern, onset, duration, quantity of bleeding, and complaints related to thyroid dysfunction, which were noted in detail. A thorough clinical examination, including a general physical examination, neck examination, and systemic and gynaecological examinations, was carried out. All patients were subjected to thyroid-stimulating hormone (TSH) and relevant investigations as indicated. Patients with abnormal thyroid profiles were subjected to triiodothyronine (T3), thyroxine (T4), and anti-TPO antibodies. 

Inclusion criteria

All women presenting to the gynaecology outpatient department/inpatient department from puberty till menopause with AUB (E/O) were included in the study.

Exclusion criteria

The exclusion criteria were as follows: patients on intrauterine contraceptive device; women with a history of liver disorders/coagulopathy; pregnant women or women with a history of childbirth in the last one year or women with a history of abortion in the last three months; associated pelvic inflammatory disease; cases refusing to participate in the study; known case of malignancy of genital organs; patients with fibroid uterus, polyp or adenomyosis.

Methodology

The study protocol included a thorough history taking regarding age, bleeding pattern, onset, duration, quantity of bleeding, and complaints related to thyroid dysfunction were noted in detail. A thorough clinical examination, including general physical examination, neck examination, systemic and gynaecologic examinations, was carried out. All patients were subjected to TSH and relevant investigations as indicated. Patients with abnormal thyroid profile, if indicated, were subjected to T3, T4 and anti-TPO antibodies.

Women aged 15-45 years presenting to the Department of Obstetrics and Gynaecology with AUB (E/O) during the study period and who provided informed consent were included in the study. Women who did not meet the predefined criteria for AUB (E/O) or who did not provide informed consent were excluded from the study.

The potential confounding variables assessed in this study included age, parity, BMI, and family history of thyroid disease. These variables were recorded during the clinical evaluation and were considered while interpreting the association between thyroid status and menstrual abnormalities. The study also recorded the presence of goitre and thyroid-related symptoms; these were considered clinical variables associated with thyroid status rather than independent confounders.

Patients were grouped into four categories: euthyroid, normal TSH levels (0.5-5 mU/ml); subclinical hypothyroidism, TSH levels between 5mU/ml-10mU/ml; hypothyroidism, TSH levels >10mU/ml; hyperthyroidism, TSH levels <0.5mU/ ml

The reference values were as follows: serum T4, 60-120 ng/ml; serum T3, 0.8 -16 ng/ml; serum TSH, 0.5-5 mU/ml.

## Results

In the analysis, 100 patients with AUB were included. The mean age of the study population was 34.85 ± 8.29 years, with a median age of 35 years (IQR, 27.50-43.00). The mean BMI was 24.74 ± 3.36 kg/m², with a median of 23.74 (IQR, 22.38-27.20). The mean TSH level was 3.89 ± 3.40, and the median was 2.78 (IQR, 1.97-4.10). The mean platelet count was 2.61 ± 0.71, with a median of 2.56 (IQR, 2.08-3.13) (Table 1). 

**Table 1:** Summary statistics of continuous variables (n=100). BMI: body mass index; TSH: thyroid-stimulating hormone

Variable	Mean ± SD	Median (IQR)	Range
Age (years)	34.85 ± 8.29	35.00 (27.50–43.00)	19.00–45.00
BMI (kg/m²)	24.74 ± 3.36	23.74 (22.38–27.20)	18.76–32.00
TSH	3.89 ± 3.40	2.78 (1.97–4.10)	0.04–15.88
Platelet count	2.61 ± 0.71	2.56 (2.08–3.13)	1.55–4.20

Among the participants, P2 was the most frequent parity category (63.0%), followed by P3 (15.0%), P1 (10.0%), P4 (7.0%), and nulliparity (5.0%). Heavy menstrual bleeding (HMB) was the most common type of AUB (40.0%), followed by oligomenorrhoea (28.0%) and amenorrhoea or frequent bleeding (16.0%). Menometrorrhagia, hypomenorrhoea, and polymenorrhoea accounted for 7.0%, 5.0%, and 4.0%, respectively. Symptoms were present for less than five months in 59.0% of the patients, for five months to one year in 38.0%, and for more than one year in 3.0% (Table 2).

**Table 2:** Distribution of categorical variables (n=100).

Variable	Category	n (%)
Parity	Nulliparous	5 (5.0%)
	Para 1	10 (10.0%)
	Para 2	63 (63.0%)
	Para 3	15 (15.0%)
	Para 4	7 (7.0%)
Type of abnormal uterine bleeding (AUB)	Heavy menstrual bleeding	40 (40.0%)
	Oligomenorrhea	28 (28.0%)
	Amenorrhea followed by excessive bleeding	16 (16.0%)
	Menometrorrhagia	7 (7.0%)
	Hypomenorrhoea	5 (5.0%)
	Polymenorrhea	4 (4.0%)
Duration of symptoms		59 (59.0%)
	Five months to one year	38 (38.0%)
	> One year	3 (3.0%)
Thyroid status	Euthyroid status	80 (80.0%)
	Subclinical hypothyroidism	10 (10.0%)
	Hypothyroidism	8 (8.0%)
	Hyperthyroidism	2 (2.0%)
AUB episode	First	81 (81.0%)
	Second	19 (19.0%)
Family history	Absent	93 (93.0%)
	Present	7 (7.0%)
Goitre	Absent	88 (88.0%)
	Present	12 (12.0%)
Thyroid symptoms	Absent	91 (91.0%)
	Present	9 (9.0%)

It was found that 80 patients (80.0%) were euthyroid, 10 (10.0%) had subclinical thyroid dysfunction, eight (8.0%) had hypothyroidism, and two (2.0%) had hyperthyroidism (Table 2). The distribution of AUB types did not differ significantly according to thyroid status (Pearson's chi-square, *p* = 0.398), and heavy menstrual bleeding remained the most frequent presentation across the thyroid-status categories (Table 3).

**Table 3:** Type of AUB according to thyroid status (Pearson's chi-square p=0.398). AUB: abnormal uterine bleeding

Type of AUB	Euthyroid, n (%)	Subclinical hypothyroidism, n (%)	Hypothyroidism, n (%)	Hyperthyroidism, n (%)	Total
Amenorrhea followed by excessive bleeding	15 (93.8%)	0 (0.0%)	0 (0.0%)	1 (6.2%)	16
Heavy menstrual bleeding	29 (72.5%)	7 (17.5%)	3 (7.5%)	1 (2.5%)	40
Hypomenorrhoea	3 (60.0%)	1 (20.0%)	1 (20.0%)	0 (0.0%)	5
Menometrorrhagia	7 (100.0%)	0 (0.0%)	0 (0.0%)	0 (0.0%)	7
Oligomenorrhoea	24 (85.7%)	1 (3.6%)	3 (10.7%)	0 (0.0%)	28
Polymenorrhea	2 (50.0%)	1 (25.0%)	1 (25.0%)	0 (0.0%)	4

No statistically significant association was observed between thyroid status and the duration of symptoms (*p* = 0.358) or AUB episodes (*p* = 0.330). Family history showed a statistically significant association with thyroid status (*p* = 0.045); however, only seven patients reported a positive family history. Strong associations were observed between thyroid status and goitre (*p* < 0.001) and between thyroid status and thyroid symptoms (*p* < 0.001) (Table 4).

**Table 4:** Clinical characteristics according to thyroid status (n=100). Pearson's chi-square test (χ^2^) was used for categorical variables. *P *< 0.05 was considered statistically significant. AUB: abnormal uterine bleeding

Variable	Category	Euthyroid n (%)	Subclinical n (%)	Hypothyroid n (%)	Hyperthyroid n (%)	χ2	*p*-value
Duration of symptoms	5 months-1 years	34 (89.5%)	3 (7.9%)	1 (2.6%)	0 (0.0%)	6.614	0.358
	< 5 months	44 (74.6%)	6 (10.2%)	7 (11.9%)	2 (3.4%)		
	> 1 year	2 (66.7%)	1 (33.3%)	0 (0.0%)	0 (0.0%)		
AUB episode	First	67 (82.7%)	7 (8.6%)	5 (6.2%)	2 (2.5%)	3.152	0.369
	Second	13 (68.4%)	3 (15.8%)	3 (15.8%)	0 (0.0%)		
Family history	Absent	77 (82.8%)	8 (8.6%)	6 (6.5%)	2 (2.2%)	9.071	0.028
	Present	3 (42.9%)	2 (28.6%)	2 (28.6%)	0 (0.0%)		
Goitre	Absent	77 (87.5%)	6 (6.8%)	4 (4.5%)	1 (1.1%)	26.255	0.000
	Present	3 (25.0%)	4 (33.3%)	4 (33.3%)	1 (8.3%)		
Thyroid symptoms	Absent	78 (85.7%)	7 (7.7%)	5 (5.5%)	1 (1.1%)	21.551	0.000
	Present	2 (22.2%)	3 (33.3%)	3 (33.3%)	1 (11.1%)		

Further, goitre was present in 12 patients overall, including four of 10 patients with subclinical thyroid dysfunction and four of eight patients with hypothyroidism. Thyroid symptoms were reported by nine patients, including three patients with subclinical dysfunction, three with hypothyroidism, and one with hyperthyroidism. These statistically significant clinical findings are presented graphically in Figure 1.

**Figure 1:**
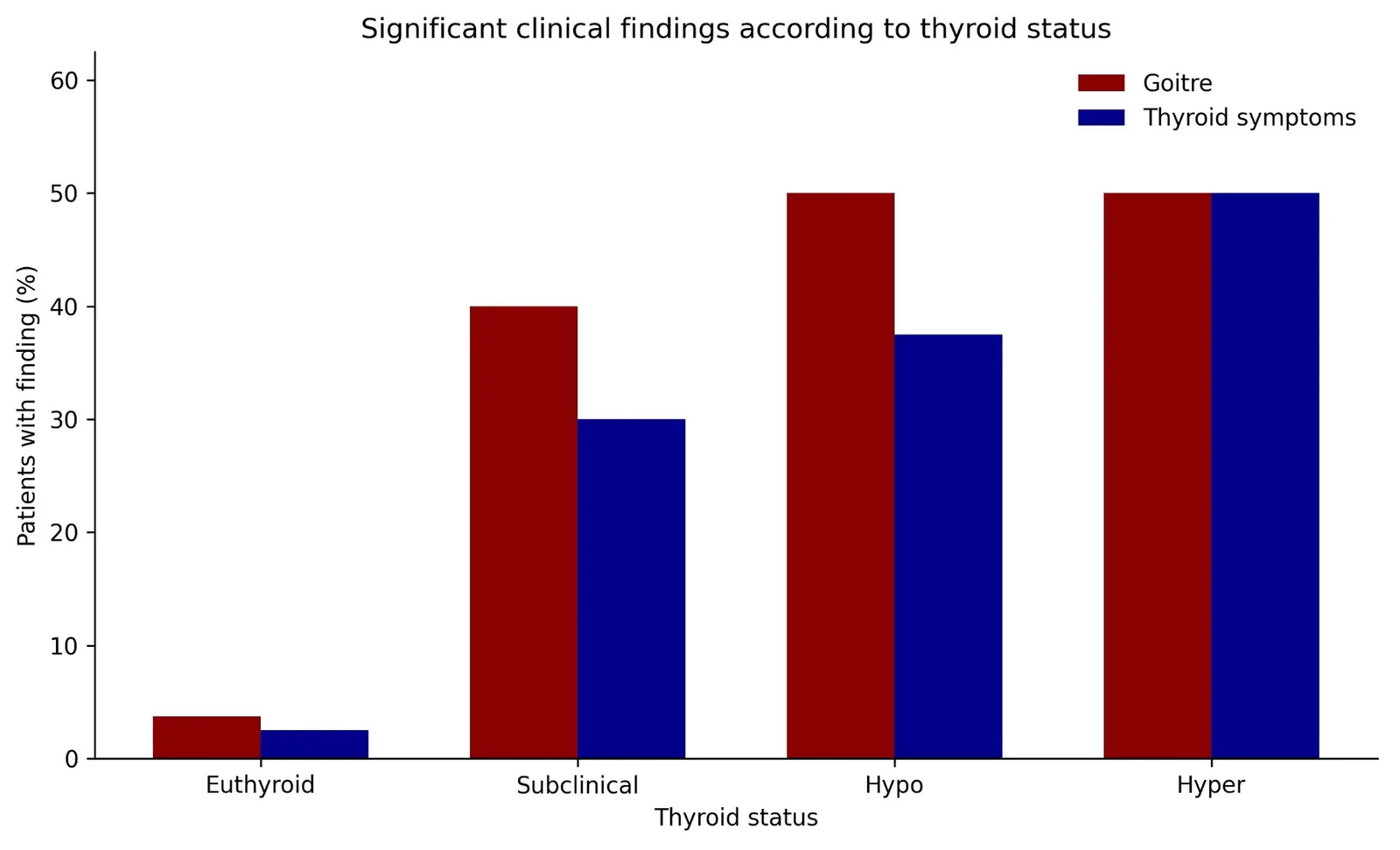
Significant clinical findings according to thyroid status. Data are presented as percentages (%) of patients within each thyroid-status group. Hypo: hypothyroid; Hyper: hyperthyroid

Through a comparison of continuous variables across the four thyroid-status groups using the Kruskal-Wallis test, it was found that age did not differ significantly (*p* = 0.264). BMI did not differ significantly across the thyroid-status groups (*p* = 0.001). As expected from the grouping variable, TSH levels differed significantly across thyroid-status categories (*p* < 0.001). Further, platelet count did not differ significantly between the thyroid- status groups (*p* = 0.837) (Table 5).

**Table 5:** Continuous variables according to thyroid status (Kruskal–Wallis test) TSH: thyroid-stimulating hormone; BMI: body mass index. Continuous variables are presented as mean ± standard deviation. The Kruskal–Wallis H test was used for comparison across thyroid-status groups. H, Kruskal–Wallis test statistic. *P* < 0.05 was considered statistically significant.

Variable	Euthyroid	Subclinical	Hypothyroid	Hyperthyroid	*p-*value
Age (years)	34.27 ± 8.42	36.00 ± 6.50	39.75 ± 8.19	32.50 ± 10.61	0.264
TSH	2.58 ± 1.10	7.59 ± 1.48	13.31 ± 1.63	0.07 ± 0.04	<0.001
BMI (kg/m²)	24.33 ± 3.36	27.07 ± 1.90	26.98 ± 2.76	20.70 ± 1.27	0.001
Platelet count	2.64 ± 0.76	2.48 ± 0.48	2.43 ± 0.48	2.83 ± 0.70	0.837

Continuous variables are presented as mean ± standard deviation, and categorical variables as frequency and percentage. Pearson chi-square tests were used for categorical associations and the Kruskal-Wallis test for continuous variables across the thyroid-status groups. A *p*-value < 0.05 was considered statistically significant.

## Discussion

Subclinical hypothyroidism was the most common abnormality identified in the present study in the clinically relevant proportion of women who presented with AUB. This is similar to the pattern observed in other studies, which have found hypothyroidism and subclinical hypothyroidism to be the most common thyroid dysfunctions among women with menstrual problems [6-10].

Bhavani et al. reported similar observations, finding thyroid abnormalities in a clinically relevant proportion of women with AUB. Pahwa et al. reported hypothyroidism in 22% and hyperthyroidism in 2% of women with dysfunctional uterine bleeding [12,13]. Other studies have also demonstrated an association between thyroid dysfunction and menstrual disorders [7,8].

Heavy menstrual bleeding was the most frequent AUB pattern found in the present study, which is consistent with observations from earlier studies. Thakur et al. reported menorrhagia in 43% of women with AUB, while previous studies have similarly described heavy bleeding as a common presentation [6,11,14]. However, an important finding in the present study was that the distribution of AUB patterns did not differ significantly according to thyroid status (*p* = 0.398). Thus, although heavy menstrual bleeding was common, this study's findings do not support attributing a particular AUB pattern specifically to thyroid dysfunction.

Moreover, the predominance of subclinical hypothyroidism is clinically relevant because mild thyroid dysfunction may occur with few or nonspecific symptoms. Wilansky and Greisman demonstrated biochemical evidence of early hypothyroidism in apparently euthyroid women presenting with menorrhagia, highlighting the possibility of occult thyroid dysfunction [10]. Joshi et al. similarly reported menstrual abnormalities among women with thyroid disease and found that menstrual disturbances preceded other clinical manifestations of thyroid dysfunction in 45% of affected women, sometimes by several months or years [15]. These observations reinforce the value of biochemical thyroid assessment even when classical thyroid symptoms are absent.

The pattern of menstrual disturbance reported in earlier thyroid studies also provides a useful context. Doifode and Fernandes reported hypothyroidism in 28.17% of women with dysfunctional uterine bleeding, with heavy menstrual bleeding occurring in 63.3% of the women with hypothyroidism [16]. Menon and Bharucha reported heavy menstrual bleeding or polymenorrhoea in 46.15% of hypothyroid women and oligomenorrhoea in 23.07%, while among hyperthyroid women, hypomenorrhoea was more frequent [17]. Likewise, Kakuno et al. demonstrated that menstrual disturbances may occur with both hypothyroidism and hyperthyroidism [5]. These reports support the biological relationship between thyroid status and menstrual function, while the nonsignificant AUB-pattern association in the present study suggests that these tendencies should not be interpreted as diagnostic patterns.

Furthermore, this study demonstrated significant associations between thyroid status and goitre and between thyroid status and thyroid-related symptoms. Goitre was present in 12 women, and thyroid-related symptoms were reported by nine women.

A positive family history was also associated with thyroid status in the present study (*p* = 0.045), although only seven women reported a positive family history. This small number limits the strength of inference and supports the interpretation of this finding as an association rather than evidence of causation. Similarly, BMI differed across the thyroid-status groups in the present dataset; however, this observation should be interpreted cautiously because of the cross-sectional design and the possibility of confounding.

The results obtained in the present study must also be seen in the context of the classification of AUB given by the FIGO PALM-COEIN algorithm [1]. Thyroid dysfunction is relevant only because of its effect on ovulatory dysfunction and thus could be one non-structural contributor to abnormal bleeding. The necessity to take other structural and non-structural aetiologies into account is reinforced by this study, as no significant correlation was found between any individual AUB pattern and thyroid status. Overall, this study's results concur with previous studies suggesting that thyroid dysfunction may be an important factor in women with AUB and especially subclinical hypothyroidism. However, no significant association between thyroid status and AUB pattern indicates that none of the bleeding patterns is predictive of thyroid dysfunction. Therefore, the thyroid should be evaluated based on the overall clinical picture and not merely the bleeding pattern.

Limitations

This study's relatively small sample size and single-centre design may limit the generalisability of the findings to other populations. Its observational, cross-sectional design does not allow causal relationships to be established between thyroid dysfunction and AUB. Further, other confounding factors, such as age, BMI, medications, and other endocrine and gynaecological conditions, could have affected the observed associations. Larger, multicentre prospective studies with longer follow-ups are needed to validate these findings and better determine the relationship between thyroid dysfunction and AUB. Patients presenting with AUB symptoms with no obvious cause should be screened for thyroid disorders. Another limitation of this study is the fact that it is confined to one centre and one country, with a small sample size.

## Conclusions

Menstrual irregularities are more common in patients with thyroid disorders and may precede the clinical diagnosis of thyroid dysfunction. Thyroid disorders should be regarded as significant etiological components, especially in cases of AUB with no obvious pathology, classified as AUB (E/O). In patients with AUB (E/O), biochemical estimation of free T3, free T4, and TSH should be considered as part of the evaluation. In many cases, appropriate treatment of thyroid disorders may help correct menstrual irregularities. Hence, screening for thyroid dysfunction should be considered in all patients presenting with menstrual irregularities, as early detection and treatment may improve clinical outcomes. Such screening may also be cost-effective in the long term by facilitating timely diagnosis and management of thyroid-related menstrual disturbances.
